# Supplementary material for: Cost-effectiveness of repeat delayed imaging for spontaneous subarachnoid hemorrhage
Source: PLoS One. 2023 Jul 26;18(7):e0289144. doi: 10.1371/journal.pone.0289144 (PMC10370759; doi:10.1371/journal.pone.0289144)
Supplement: S1 Fig — (PDF) [file pone.0289144.s001.pdf]

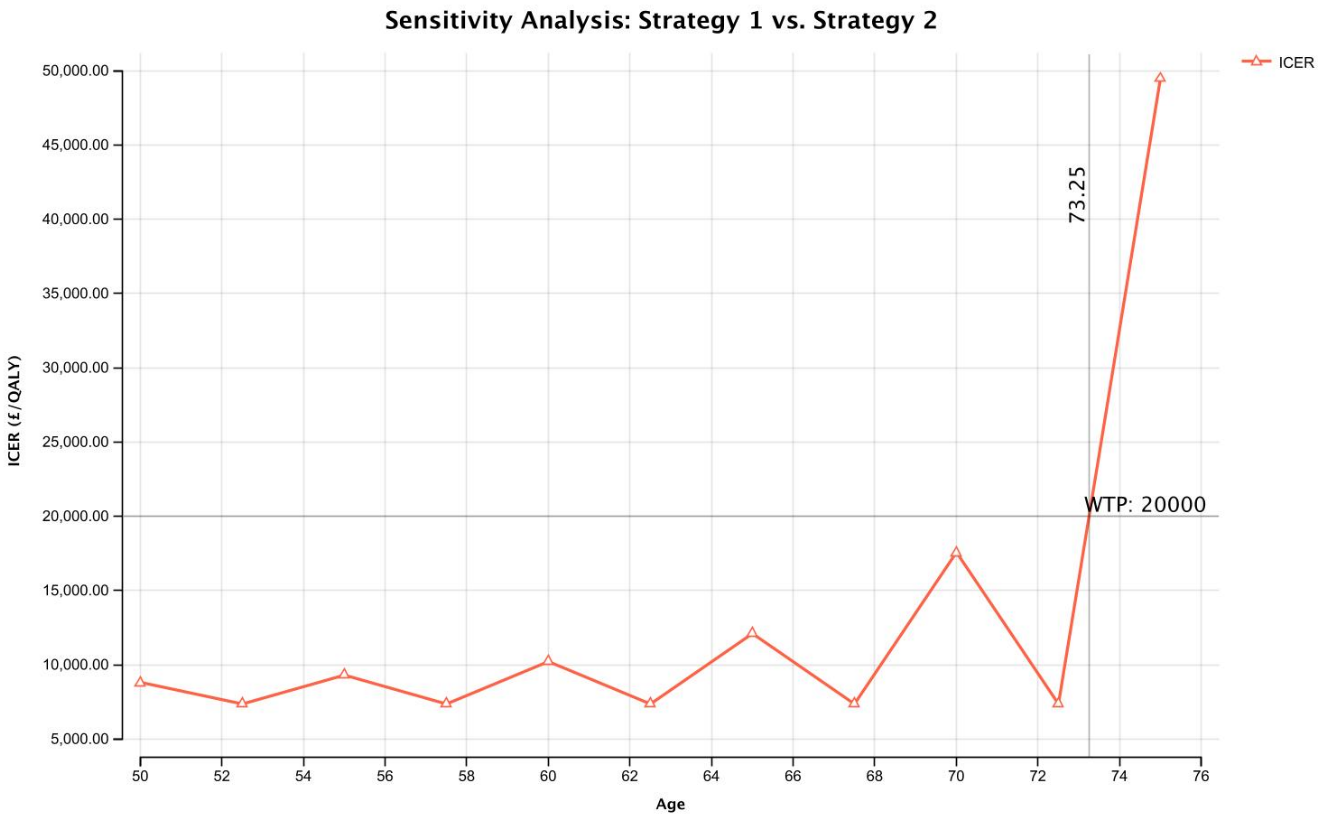

Fig 1. Results of one-way sensitivity analysis for Age. This analysis was performed by using a WTP of £20 000/ QALY. ICER, incremental cost-effectiveness ratio.

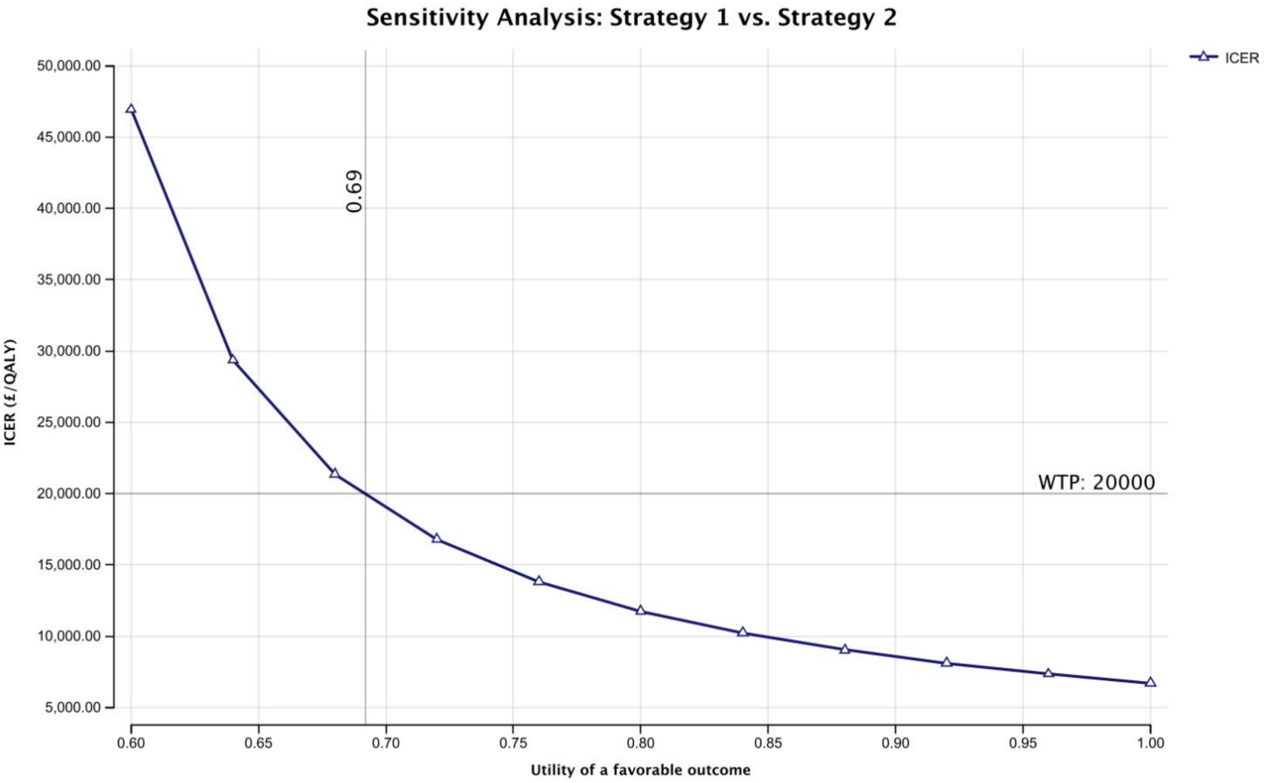

Fig 2. Results of one-way sensitivity analysis for Utility of a favorable outcome. This analysis was performed by using a WTP of £20 000/ QALY. CTA, computed tomographic angiography; ICER, incremental cost-effectiveness ratio.

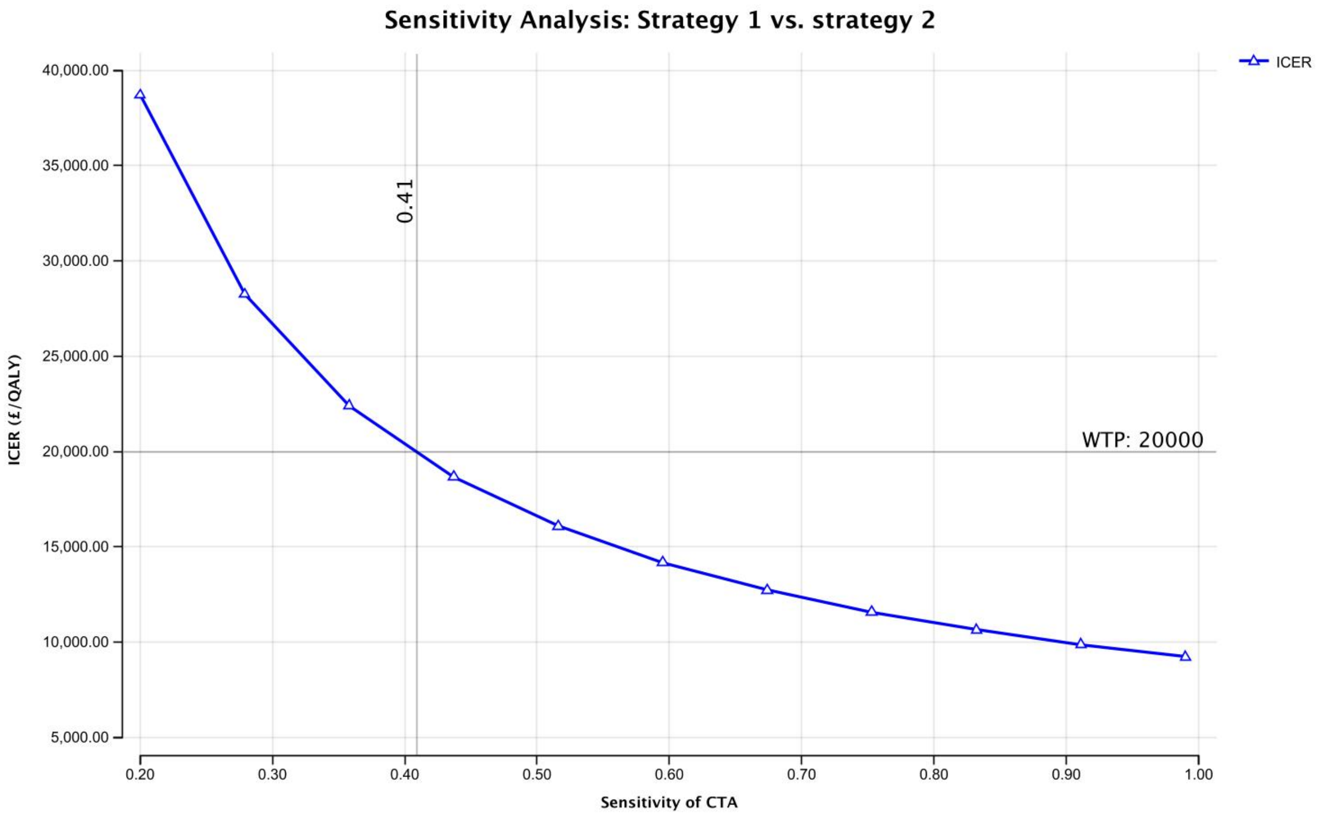

Fig 3. Results of one-way sensitivity analysis for Sensitivity of CTA. This analysis was performed by using a WTP of £20 000/ QALY. CTA, computed tomographic angiography; ICER, incremental cost-effectiveness ratio.

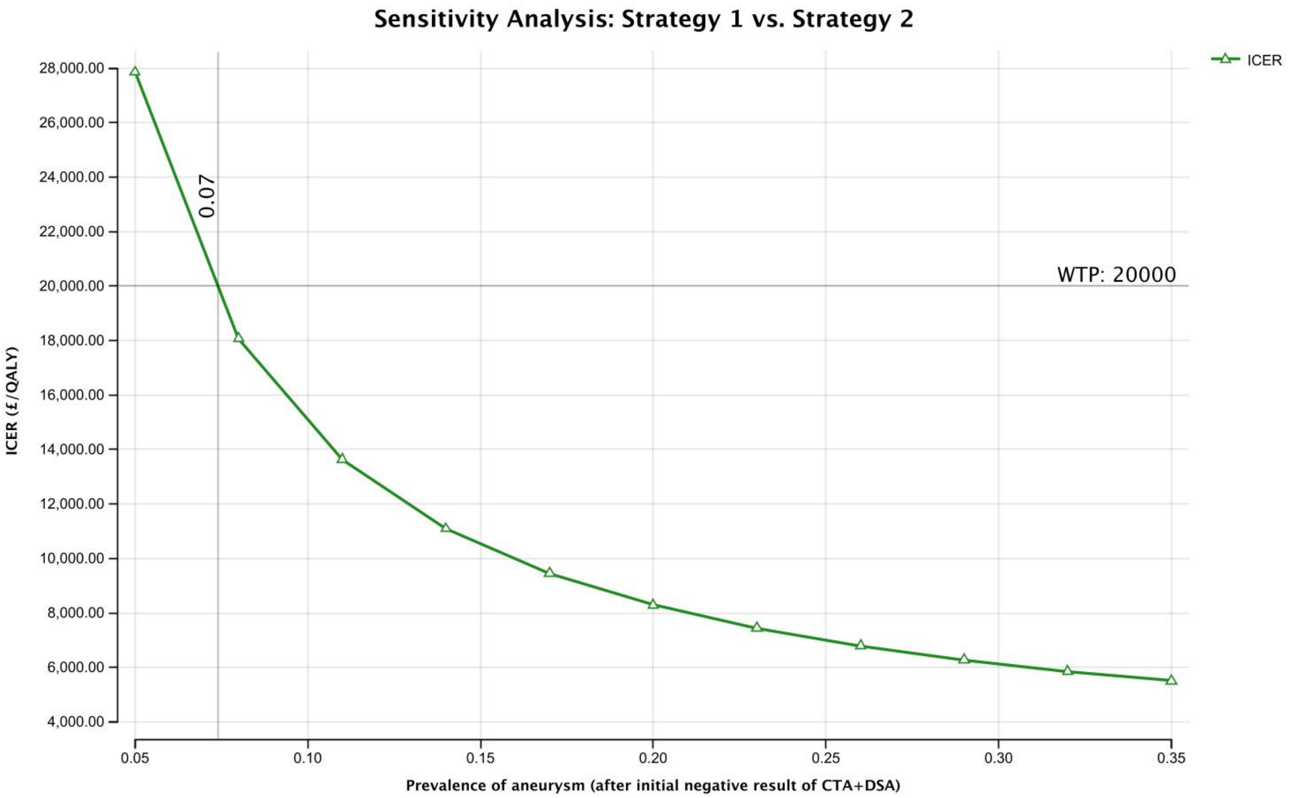

Fig 4. Results of one-way sensitivity analysis for Incidence of aneurysm. This analysis was performed by using a WTP of £20 000/ QALY. CTA, computed tomographic angiography; ICER, incremental cost-effectiveness ratio.
