# Supplementary material for: Cost-effectiveness of repeat delayed imaging for spontaneous subarachnoid hemorrhage
Source: PLoS One. 2023 Jul 26;18(7):e0289144. doi: 10.1371/journal.pone.0289144 (PMC10370759; doi:10.1371/journal.pone.0289144)
Supplement: S1 Table — (DOCX) [file pone.0289144.s002.docx]

1. The rate of death for aSAH after treatment [^[[1]](#endnote-0)^]
2. The rate of favorable outcome for aSAH after treatment ^[1]^
3. Annual probability of re-bleeding and incidence of fatal re-bleeding for untreated aneurysm (10-year)[^[[2]](#endnote-1)^]

**Table 1. Annual probability of health outcome for aSAH after treatment**

| Year | Annual mortality rate (%)( coiling) | Annual mortality rate (%)  (clipping) | Annual probability of favorable outcome after treated aneurysm (%) ( coiling) | Annual probability of favorable outcome after treated aneurysm (%) ( clipping) |
| --- | --- | --- | --- | --- |
| 1 | 6.94 | 10.00 | 75.84 | 75.10 |
| 2 | 7.32 | 11.43 | 77.02 | 77.87 |
| 3 | 8.28 | 13.61 | 76.07 | 80.53 |
| 4 | 9.33 | 14.55 | 74.24 | 80.06 |
| 5 | 10.17 | 15.36 | 73.49 | 81.37 |
| 6 | 12.26 | 17.15 | 73.82 | 81.12 |
| 7 | 14.43 | 20.06 | 71.99 | 80.45 |
| 8 | 16.24 | 22.21 | 69.11 | 79.89 |
| 9 | 18.33 | 24.70 | 68.18 | 80.63 |
| 10 | 19.85 | 27.13 | 66.03 | 78.66 |

| Annual probability of all re-bleeding for SAH patients | | Annual probability of fatal re-bleeding for SAH patients | |
| --- | --- | --- | --- |
| Year | Incidence (%) | Year | Incidence (%) |
| 1 | 2.3 | 1 | 1.9 |
| 2 | 3.1 | 2 | 2.0 |
| 3 | 5.9 | 3 | 3.7 |
| 4 | 4.7 | 4 | 2.8 |
| 5 | 3.8 | 5 | 2.4 |
| 6 | 3.7 | 6 | 2.0 |
| 7 | 3.5 | 7 | 2.3 |
| 8 | 5.3 | 8 | 1.8 |
| 9 | 3.9 | 9 | 3.1 |
| 10 | 0 | 10 | 0 |
| total | 36.2 | total | 22 |

**Table 2. Annual probability of re-bleeding and fatal re-bleeding for untreated aneurysm**

**References**

1. [].Hua X, Gray A, Wolstenholme J, Clarke P, Molyneux AJ, Kerr RSC, et al. Survival, Dependency, and Health-Related Quality of Life in Patients With Ruptured Intracranial Aneurysm: 10-Year Follow-up of the United Kingdom Cohort of the International Subarachnoid Aneurysm Trial. Neurosurgery. 2021;13;88(2):252-260. [↑](#endnote-ref-0)
2. [].Winn HR, Richardson AE, Jane JA. The long-term prognosis in untreated cerebral aneurysms: I. The incidence of late hemorrhage in cerebral aneurysm: a 10-year evaluation of 364 patients. Ann Neurol. 1977;1(4):358-70. [↑](#endnote-ref-1)
