# Supplementary material for: Cost-effectiveness of repeat delayed imaging for spontaneous subarachnoid hemorrhage
Source: PLoS One. 2023 Jul 26;18(7):e0289144. doi: 10.1371/journal.pone.0289144 (PMC10370759; doi:10.1371/journal.pone.0289144)
Supplement: Attachment — Submitted filename: ONE-D-22-31987-recommendation.docx [file pone.0289144.s003.docx]

**Title: Cost effectiveness of repeat delayed imaging for spontaneous subarachnoid**

**hemorrhage**

**Manuscript Number: PONE-D-22-31987**

Comments:

This study evaluated the cost-effectiveness of repeat delayed imaging in patients with

SAH who had a negative result during their initial imaging from a healthcare perspective.

The manuscript was very well-written and logically constructed.

I totally agree to accept for publication, while some minor revisions are needed.

1. In abstract, the control group should be clarified in the description of results.
2. Some minor grammar errors need to be revised, for example, in line 110 should say ‘were’ instead of ‘was’.
3. The time horizon ‘lifetime’ may need to be clarified in methods section.
4. Complications for patients with SAH were not explicitly modeled in this study, the authors have illustrated this as a limitation, this is acceptable.
5. Line 130-136: Sensitivity Analysis: the range of sensitivity and specificity should be clarified.

Publication criteria:

1. The study presents the results of original research.

Yes
2. Results reported have not been published elsewhere.

Yes
3. Experiments, statistics, and other analyses are performed to a high technical standard and are described in sufficient detail.

Yes
4. Conclusions are presented in an appropriate fashion and are supported by the data.

Yes
5. The article is presented in an intelligible fashion and is written in standard English.

Yes
6. The research meets all applicable standards for the ethics of experimentation and research integrity.
7. The article adheres to appropriate reporting guidelines and community standards for data availability.

Yes
