# Supplementary material for: Cost-effectiveness of repeat delayed imaging for spontaneous subarachnoid hemorrhage
Source: PLoS One. 2023 Jul 26;18(7):e0289144. doi: 10.1371/journal.pone.0289144 (PMC10370759; doi:10.1371/journal.pone.0289144)
Supplement: Attachment — Submitted filename: Response to Reviewers.docx [file pone.0289144.s004.docx]

**Response Letter**

On behalf of my co-authors, we thank you very much for giving us the opportunity to revise our manuscript. We appreciate the editor and all the reviewers very much for their invaluable feedback, positive remarks, and constructive suggestions provided on our manuscript entitled "Cost-effectiveness of repeat delayed imaging for spontaneous subarachnoid hemorrhage" (PONE-D-22-31987). We have carefully considered each of the reviewers' comments and have made revisions, which are clearly marked in blue in the paper.

**Journal requirements:**

When submitting your revision, we need you to address these additional requirements.

1. Please ensure that your manuscript meets PLOS ONE's style requirements, including those for file naming. The PLOS ONE style templates can be found at

<https://journals.plos.org/plosone/s/file?id=wjVg/PLOSOne_formatting_sample_main_body.pdf> and

<https://journals.plos.org/plosone/s/file?id=ba62/PLOSOne_formatting_sample_title_authors_affiliations.pdf>.

**Response:** Thanks for your suggestion. We have amended our manuscript and file naming to meet PLOS ONE's style requirements.

2. We noticed you have some minor occurrence of overlapping text with the following previous publication(s), which needs to be addressed:

- https://doi.org/10.1161/CIRCOUTCOMES.119.006406

In your revision ensure you cite all your sources (including your own works), and quote or rephrase any duplicated text outside the methods section. Further consideration is dependent on these concerns being addressed.

**Response:** We sincerely appreciate your suggestion and have checked the similarities between our paper and other published papers using 'iThenticate.' We can assure you that any overlapping text has been rewritten, and all relevant references have been added. After the revision, the similarity score of our paper has dropped to 18% (based on the iThenticate plagiarism checker).

3. Please review your reference list to ensure that it is complete and correct. If you have cited papers that have been retracted, please include the rationale for doing so in the manuscript text, or remove these references and replace them with relevant current references. Any changes to the reference list should be mentioned in the rebuttal letter that accompanies your revised manuscript. If you need to cite a retracted article, indicate the article’s retracted status in the References list and also include a citation and full reference for the retraction notice.

**Response:** As advised, we have carefully reviewed our reference list and ensure that it is complete and correct.

The changes made to the reference list are summarized as follows:

- The original reference: “*1. Macdonald RL, Schweizer TA. Spontaneous subarachnoid haemorrhage. Lancet. 2017;389(10069):655-666.*” has been updated to “*1. Claassen J, Park S. Spontaneous subarachnoid haemorrhage. Lancet. 2022;400(10355):846-862.*”, and reference “*2. GBD 2019 Stroke Collaborators. Global, regional, and national burden of stroke and its risk factors, 1990-2019: a systematic analysis for the Global Burden of Disease Study 2019. Lancet Neurol. 2021;20(10):795-820*.” has been added to provide the latest evidence and substantiate the current state of Subarachnoid hemorrhage (SAH).
- The reference: “*8. Brown RD Jr, Broderick JP. Unruptured intracranial aneurysms: epidemiology, natural history, management options, and familial screening. Lancet Neurol. 2014;13(4):393-404.*” has been added to provide the latest epidemiological evidence on unruptured intracranial aneurysms.
- The original reference: “*8. van Gijn J, Rinkel GJ. Subarachnoid haemorrhage: diagnosis, causes and management. Brain. 2001;124(Pt 2):249-278*.” has been updated to “*10. Tawk RG, Hasan TF, D'Souza CE, Peel JB, Freeman WD. Diagnosis and Treatment of Unruptured Intracranial Aneurysms and Aneurysmal Subarachnoid Hemorrhage. Mayo Clin Proc. 2021;96(7):1970-2000.*” to provide the latest evidence.
- The reference: *“22. Siebert U, Alagoz O, Bayoumi AM, Jahn B, Owens DK, Cohen DJ, et al. State-transition modeling: a report of the ISPOR-SMDM modeling good research practices task force-3. Value in Health, 2012, 15(6): 812-820.*” has been added to clarify the reference sources for determining the time period of the model.
- The reference: “*23. Jethwa PR, Gandhi CD, Prestigiacomo CJ. Cost-effectiveness of computed tomographic angiography in screening for aneurysm in spontaneous subarachnoid hemorrhage. Neurosurgery, 2014, 61: 137-144.*” has been added also to clarify the reference sources for determining the time period of the model.
- The reference: “*36. Borgonovo E, Plischke E. Sensitivity analysis: A review of recent advances. European Journal of Operational Research, 2016, 248(3): 869-887.*” has been added to provide the sources for interpreting the Tornado diagram.
- The reference: “*37. The Organisation for Economic Co-operation and Development (OECD) Health Statistics 2022. Available from: https://www.oecd.org/health/health-data.htm.*” has been added to provide the latest health spending evidence.
- The reference: “38. *National Institute for Health and Care Excellence (NICE) guideline [NG228] 2022. Subarachnoid haemorrhage caused by a ruptured aneurysm: diagnosis and management. Available from: https://www.nice.org.uk/guidance/ng228/chapter/Recommendations#monitoring-and-managing-complications*” has been added to provide the latest evidence aimed at improving the quality of life for survivors of aneurysms.
- The reference: “39. *Bottinor W, Polkampally P, Jovin I. Adverse Reactions to Iodinated Contrast Media. Int J Angiol. 2013:149–154*” and “*40. Rao QA, Newhouse JH. Risk of nephropathy after intravenous administration of contrast material: a critical literature analysis. Radiology. 2006;239:392–397*.” have been added to further supplement the existing evidence regarding the potential complications of CTA.

**Additional Editor Comments:**

Thank you Dr. Jin et al. The manuscript nicely models a cost-effectiveness of imaging. I would recommend modifications according to the attached reviewer comments.

**Response:** We appreciate your positive remarks regarding our paper. We have carefully reviewed the attached reviewer comments and addressed them one-by-one.

**Responds to the reviewer’s comments**

**Reviewer #1**: This study evaluated the cost-effectiveness of repeat delayed imaging in patients with SAH who had a negative result during their initial imaging from a healthcare perspective.

The manuscript was very well-written and logically constructed.

I totally agree to accept for publication, while some minor revisions are needed.

**Response:** Thank you for your positive feedback on our manuscript. We are delighted to hear that you found the study well-written and logically constructed. We have carefully addressed the revisions suggested by you to further enhance the quality and clarity of the paper.

1. In abstract, the control group should be clarified in the description of results.

**Response:** Thanks for your suggestion. Information about control group has been added to the results section of the abstract:

**Line 12-13:** “The base case incremental cost-effectiveness ratio (ICER) of repeat delayed imaging is £9,314 per QALY **compared to no-repeat delayed imaging**.”

2. Some minor grammar errors need to be revised, for example, in line 110 should say ‘were’ instead of ‘was’.

**Response:** Thanks for spotting this mistake. As suggested, "was" has been corrected to "were" **(Line 110**).

3. The time horizon ‘lifetime’ may need to be clarified in methods section.

**Response:** Thanks for your suggestion. The following sentence has been added to the Methods section in the main text to clarify the time horizon ‘lifetime’.

**Line 59-60**: “A lifetime horizon was adopted for the cost-effectiveness analysis, which means that the patients' health outcomes and costs were calculated until their death.”

4. Complications for patients with SAH were not explicitly modeled in this study, the authors have illustrated this as a limitation, this is acceptable.

**Response:** Thank you for your understanding. The following sentence has been added to the Discussion section to discuss the implications of omitting complications of CTA on the results:

**Line 253-259**: “Second, the potential complications of CTA, such as acute allergic reactions and contrast-induced nephropathy, were not included in our model due to a lack of long-term cost and health outcome data for patients experiencing those complications. However, existing evidence suggests that the risk of severe allergic reactions and contrast-induced nephropathy is extremely low or even negligible for patients who undergo CTA [39,40]. Therefore, we do not anticipate that the omission of these complications would significantly alter the conclusions of our study.”

References:

[39] Bottinor W, Polkampally P, Jovin I. Adverse Reactions to Iodinated Contrast Media. Int J Angiol. 2013:149–154.

[40] Rao QA, Newhouse JH. Risk of nephropathy after intravenous administration of contrast material: a critical literature analysis. Radiology. 2006;239:392–397.

5. Line 130-136: Sensitivity Analysis: the range of sensitivity and specificity should be clarified.

**Response:** Thanks for your suggestion. The ranges of sensitivity (20.00% ~ 99.00%) and specificity of DSA (20.00% ~ 100.00%) that we tested in sensitivity analysis are reported in Table 1. The following sentence has been added to the Methods section to explain how the range of sensitivity analysis was determined (**Line 141-143**): “The range for sensitivity analysis was established by considering the upper and lower values of the variable as reported in the published literature.”

**Responds to the reviewer’s comments**

**Reviewer #2**: The authors investigated the cost effectiveness of using repeat delayed imaging to diagnosis unruptured aneurysms that were missed after initial imaging. To do so, the authors developed a Markov model using available data relating to costs of imaging and treatment in the UK and the utility of certain outcomes. The authors presented their results as they related to the lifetime costs and QALY that patients were estimated to experience. This paper was well-written and easily digestible; however, the below points should be considered before the work is published.

**Response:** Thanks for recognizing the importance of our work and for dedicating your time to reviewing and improving this paper. We have carefully addressed each of the points that you made and incorporate any necessary revisions to further improve the quality and accuracy of our work.

1. Given that the model was specifically designed using assumptions for 55-year-old patients, it would be prudent for the authors to explicitly note this limitation in the ability to generalize their findings to a more diverse patient population.

**Response:** Thanks for your valuable comment. To test the impact of patient age on the cost-effectiveness results, we tested a range of different ages (50-80 years) and found that for patients over 73 years old, repeat delayed imaging using CTA is no longer cost-effective. The sensitivity analysis result of age has been added to the tornado diagram (Fig 2, see below). In addition, the following changes have been made to the manuscript:

- Abstract (**Line 16-17**): “The results are sensitive to age, the utility of survived patients with a favorable outcome…”
- Result section (**Line 168-171 & Fig.2**): “The base case conclusion (repeat delayed imaging using CTA being the most cost-effective intervention) is sensitive to the following parameters: age of the patient increased to 73.25 years old (base case value: 55 years old) …...”
- Discussion section (**Line 194-197)**: “The cost-effectiveness of the repeat delayed imaging strategy was most sensitive to age, the utility of favorable outcomes, the sensitivity of CTA, and ……”
- Discussion section (**Line 231-232)**: “These findings suggest that repeat delayed imaging might not be cost-effective for patients over 73 years old.”

Fig. 2 A tornado diagram of 1-way sensitivity analyses to demonstrate the effects of varying parameters on the incremental cost-effectiveness ratio (ICER) for No second-line strategy vs Delayed CTA imaging strategy. The wider bars at the top have the greatest effect on the ICER, while variations in inputs at the bottom have small effects. The willingness-to-pay (WTP) line is at an ICER of £20,000. Numbers in parentheses after the variables are the parameter ranges. CTA, computed tomographic angiography; DSA, digital subtraction angiography; ICER, incremental cost-effectiveness ratio; QALY, quality-adjusted life-year.

2. Why did the authors choose to divide the time period of the study into one year cycles? A brief description of the reasoning and a relevant citation would help ground this decision.

**Response:** Thanks for your valuable advice. The following justification for adopting a one-year cycle has been added to the main text (**Line 85-91**): “The time period of the model is divided into cycles of time. After discussion with clinicians, a one-year cycle length was adopted for this study considering the growth rate of SAH, frequency of re-bleeding, the time interval adopted by the long-term follow-up studies, and computational burden of the model [22]. A one-year cycle length was also adopted by previous economic analysis assessing similar decision question [23]. At the end of each one-year cycle in the Markov process, individuals could either transition to a different health state or continue residing in their current state.”

References:

[22] Siebert U, Alagoz O, Bayoumi A M, et al. State-transition modeling: a report of the ISPOR-SMDM modeling good research practices task force-3[J]. Value in Health, 2012, 15(6): 812-820.

[23] Jethwa P R, Gandhi C D, Prestigiacomo C J. Cost-effectiveness of computed tomographic angiography in screening for aneurysm in spontaneous subarachnoid hemorrhage[J]. Neurosurgery, 2014, 61: 137-144.

3. The authors do not specify what aneurysm treatment method was used in the model (i.e. clipping versus coiling), which would be helpful in determining the overall applicability of their findings. Were both treatment methods accounted for or only one? If only one was factored into the analysis, this limitation should be noted.

Response: Thank you for your comment. We would like to clarify that both clipping and coiling were considered in our model. In the base case analysis, we assumed that patients received coiling, while in the sensitivity analysis, we tested the alternative assumption that patients received clipping. The results of the sensitivity analysis, as shown in Figure 2 below, indicate that switching from coiling to clipping had a minor impact on the cost-effectiveness results.

Fig. 2 A tornado diagram of 1-way sensitivity analyses to demonstrate the effects of varying parameters on the incremental cost-effectiveness ratio (ICER) for No second-line strategy vs Delayed CTA imaging strategy. The wider bars at the top have the greatest effect on the ICER, while variations in inputs at the bottom have small effects. The willingness-to-pay (WTP) line is at an ICER of £20,000. Numbers in parentheses after the variables are the parameter ranges. CTA, computed tomographic angiography; DSA, digital subtraction angiography; ICER, incremental cost-effectiveness ratio; QALY, quality-adjusted life-year.

4. The tornado diagram was difficult to understand; a more detailed explanation of how to interpret the diagram would be helpful in parsing the key points, as well as a higher-resolution image.

**Response:** We gratefully appreciate your suggestion. As suggested, a higher-resolution image has been provided, shown in the above Figure 2. In addition, a more detailed explanation of how to interpret the tornado diagram has been added to the main text:

**Line 144-149**: “The Tornado diagram in Figure 2 showed the relative importance of key parameters on the ICER. The horizontal bars in the diagram represent the range of each parameter. For example, the range of the utility of a favorable outcome is 0.6-1. The width of each bar indicates the magnitude of its impact on the ICER – the longer the bar, the greater influence the parameter has on ICER. As shown in Figure 2, the top four most influential variables to our model are age, the utility of a favorable outcome, sensitivity of CTA, and prevalence of aneurysm.”

5. The authors mention two studies that were conducted in the US (Jethwa et al. and Kalra et al.) but do not comment on the fundamental differences in healthcare systems and overall healthcare costs between the two countries; the authors should consider briefly discussing these differences in the estimated costs used in the US studies versus their own UK study and how these differences, if any, may account for the difference in their findings.

**Response:** Thanks for your valuable comment. The following sentences have been changed in the main text:

**Line 206-220**: “This might be because of three reasons. Firstly, Jethwa *et al.* used CTA as the first-line test and DSA as the second-line test, whilst our study used DSA+CTA as the first-line test and CTA as the second-line test. Secondly, both Jethwa *et al.* and Kalra *et al* adopted a short time horizon (i.e., only covered the time period from patients receiving the initial imaging to patients receiving a definitive diagnosis), whilst our study adopted a lifetime horizon. Therefore, the two US studies are likely to underestimate the QALY losses and future treatment costs for those patients with aneurysms who were missed by the initial imaging. If those lifetimes QALY losses and future treatment costs were considered, repeat delayed imaging would appear to be more cost-effective. Last but not least, due to the difference in financing mechanisms of healthcare system, payment structure and approaches to healthcare delivery, the price of healthcare services and procedures in the US is consistently higher than the price in the UK. According to the OECD Health Statistics 2022, the per capita health spending in the U.S. was more than two times higher than other developed countries, including the UK, Australia, France, Canada and New Zealand [37]. As a result, the cost-effectiveness results of studies conducted in the US might not be transferable to the UK setting.

Reference:

[37]The Organisation for Economic Co-operation and Development (OECD) Health Statistics 2022. Available from: https://www.oecd.org/health/health-data.htm.

6. Additionally, the authors note that one of the limitations of their study includes not explicitly modeling complications due to a lack of evidence. It is not clear to me what is meant by "lack of evidence". Are the authors referring to a lack of available data about the cost of complications from SAH in the UK? Given that Jethwa et al. includes complication-associated costs in their analysis, it may be prudent to explain in more detail how this limitation came about and the possible effect its exclusion may have on the results.

**Response:** Thanks for your question. The following sentence has been added to the Discussion section to clarify our intended meaning behind the phrase "lack of evidence" and to address the implications of omitting complications on the results:

**Line 253-259**: “Second, the potential complications of CTA, such as acute allergic reactions and contrast-induced nephropathy, were not included in our model due to a lack of long-term cost and health outcome data for patients experiencing those complications. However, existing evidence suggests that the risk of severe allergic reactions and contrast-induced nephropathy is extremely low or even negligible for patients who undergo CTA [39,40]. Therefore, we do not anticipate that the omission of these complications would significantly alter the conclusions of our study.”

References:

[39] Bottinor W, Polkampally P, Jovin I. Adverse Reactions to Iodinated Contrast Media. Int J Angiol. 2013:149–154.

[40] Rao QA, Newhouse JH. Risk of nephropathy after intravenous administration of contrast material: a critical literature analysis. Radiology. 2006;239:392–397.

7. Finally, a more detailed explanation of how to interpret the utility and mortality of functional outcome would aid in understanding the results of the model and its sensitivity. For example, what factors may influence "the utility of survived patients with a favorable outcome"?

**Response:** Thanks for your valuable advice. The following sentence has been added to the Discussion section to explain what strategies can be used to improve the utility of survived patients with a favorable outcome and thus makes repeat delayed imaging more cost-effective:

**Line 233-236**: “Potential strategies to improve the quality of life for aneurysm survivors include developing a follow-up care plan with the patient, providing rehabilitation services, monitoring blood pressure and headaches, and promoting a healthy life-style that includes abstaining from smoking and engaging in regular exercise [38].”

Reference:

[38] National Institute for Health and Care Excellence (NICE) guideline [NG228] 2022. Subarachnoid haemorrhage caused by a ruptured aneurysm: diagnosis and management. Available from: https://www.nice.org.uk/guidance/ng228/chapter/Recommendations#monitoring-and-managing-complications.
